# 2-(2-Methyl-2-nitrovinyl)furan but Not Furvina Interfere with *Staphylococcus aureus* Agr Quorum-Sensing System and Potentiate the Action of Fusidic Acid against Biofilms

**DOI:** 10.3390/ijms22020613

**Published:** 2021-01-09

**Authors:** Diana Oliveira, Anabela Borges, Reinaldo Molina Ruiz, Zenaida Rodríguez Negrín, Simona Distinto, Fernanda Borges, Manuel Simões

**Affiliations:** 1 LEPABE–Laboratory for Process Engineering, Environment, Biotechnology and Energy, Department of Chemical Engineering, Faculty of Engineering, University of Porto, 4200-465 Porto, Portugal; up201406841@fe.up.pt (D.O.); apborges@fe.up.pt (A.B.); 2 CIQUP—Research Center in Chemistry, Department of Chemistry and Biochemistry, Faculty of Sciences, University of Porto, 4169-007 Porto, Portugal; fborges@fc.up.pt; 3 Centro de Bioactivos Quimico, Universidad Central de las Villas, 54830 Santa Clara, Cuba; reymolina@uclv.edu.cu (R.M.R.); zenaidar@uclv.edu.cu (Z.R.N.); 4 Department of Life and Environmental Sciences, University of Cagliari, Via Ospedale 72, 09124 Cagliari, Italy; s.distinto@unica.it

**Keywords:** Furvina, 2-nitrovinylfuran derivatives, *Staphylococcus aureus*, quorum-sensing inhibition, biofilms, antimicrobial combination, antimicrobial resistance

## Abstract

Quorum sensing (QS) plays an essential role in the production of virulence factors, in biofilm formation and antimicrobial resistance. Consequently, inhibiting QS is being considered a promising target for antipathogenic/anti-virulence therapies. This study aims to screen 2-nitrovinylfuran derivatives structurally related to Furvina (a broad-spectrum antibiotic already used for therapeutic purposes) for their effects on QS and in biofilm prevention/control. Furvina and four 2-nitrovinylfuran derivatives (compounds **1**–**4**) were tested to assess the ability to interfere with QS of *Staphylococcus aureus* using bioreporter strains (*S. aureus* ALC1742 and ALC1743). The activity of Furvina and the most promising quorum-sensing inhibitor (QSI) was evaluated in biofilm prevention and in biofilm control (combined with fusidic acid). The biofilms were further characterized in terms of biofilm mass, viability and membrane integrity. Compound **2** caused the most significant QS inhibition with reductions between 60% and 80%. Molecular docking simulations indicate that this compound interacts preferentially with the protein hydrophobic cleft in the LytTR domain of AgrA pocket. Metabolic inactivations of 40% for *S. aureus* ALC1742 and 20% for *S. aureus* ALC1743 were reached. A 24 h-old biofilm formed in the presence of the QSI increased the metabolic inactivation by fusidic acid to 80%, for both strains. The overall results highlight the effects of compound **2** as well as the potential of combining QSI with in-use antibiotics for the management of skin and soft tissues infections.

## 1. Introduction

*Staphylococcus aureus* is an opportunistic pathogen widely associated with skin colonization and biofilm-related infections [1]. The skin represents the first defense line of the human body against the invasion of pathogenic microorganisms. The formation of a fissure may allow the colonization by pathogens and lead to skin and soft tissue infections (SSTI). Usually, the treatment of these infections involves the administration of a topical antibiotic or antiseptics, depending on the severity of the clinical manifestation [2]. However, due to the widespread use of topical antibiotics, particularly mupirocin and fusidic acid, there is an increase in bacterial resistance [2]. Such phenomenon is closely associated with ability of bacteria to adapt to the environment they inhabit, either by changing their phenotype or their metabolism [3]. Besides that, bacteria possess a gene regulation mechanism known as quorum sensing (QS) that is used to orchestrate group behavior [4]. This mechanism is based on intercellular communication and occurs only when bacteria reach a threshold cell density. This process of chemical communication involves the production, release and detection of extracellular signaling molecules called autoinducers (AIs) [5]. In Gram-negative bacteria the AIs are usually *N*-acyl-homoserine lactones (HSLs), while in Gram-positive they are generally peptides (AIP) [6]. In *S. aureus*, the QS system is designated as accessory gene regulator (Agr) as it is encoded by *agr* locus. The *agr* locus consists of two divergent transcriptional units, RNAII and RNAIII, which are driven by P2 and P3 promoters, respectively [7]. The RNAII transcript contains a four set of genes, Agr*BDCA*, that are involved in the successful run of the Agr QS circuit [7]. AgrD is the precursor peptide of AIP, and AgrB is involved in generating and exporting the mature AIP [5,8]. When AIP accumulates outside the cell, a critical concentration is reached and it binds to a surface receptor, the transmembrane protein AgrC [7]. AgrC is a two-component sensor histidine kinase that is activated upon binding to AIP, and from this reaction a phosphate is transduced to AgrA [5]. AgrA activates the expression of P2 and P3 which will induce transcription of RNAII and RNAIII, respectively [8]. RNAIII, the effector molecule of Agr, is also responsible for upregulating the expression of extracellular virulence factors, such as toxins and enterotoxins. Thus, contributing for bacterial pathogenicity [9,10].

Associated with *S. aureus* Agr QS system is biofilm formation. In fact, Agr affects not only biofilm formation but also its maturation [11]. Biofilm is a complex process that is not only mediated by QS, but it also combines mechanisms of adhesion, synthesis of extracellular polymeric substances (EPS) and bacterial motility [12,13,14]. So far, it has been showed that the effect of QS in *S. aureus* biofilm maturation is mostly due to the decreased expression of detachment molecules, such as δ-toxin [15]. However, other molecules are downregulated by Agr and, as such, they are only expressed in an early stage of biofilm formation [11]. This offers an attractive strategy to treat *S. aureus* infections by modulating QS transcriptional regulators to prevent biofilm formation and inhibit virulence factors [16,17]. In fact, the use of QS inhibitors (QSIs) is receiving great attention and diverse studies have been developed regarding this topic—using QSIs either as anti-biofilm agents or as adjuvants to antimicrobials [18,19,20].

Due to the current limitations of antimicrobial drug discovery programs, a particular focus on repurposing and repositioning of drugs or drug candidates has been given for finding new therapeutic applications [21,22,23]. Furvina is a nitrovinylfuran ((*Z*)-2-bromo-5-(2-bromo-2-nitrovinyl)furan), synthesized from furfural a starting material present in sugar cane bagasse, reported to act as antimicrobial for topical application. Furvina is described to be a broad-spectrum antibiotic and is commercialized in Cuba as Vitrofural^®^, Furvinol^®^ and Dermofural^®^ [24,25]. The mode of action of Furvina was previously described by Frabetti et al., where they demonstrated antibacterial properties through the inhibition of protein synthesis [24]. Furthermore, these authors concluded that Furvina targets the P-site of the 30S subunit and inhibits the translation inititation pathway [24]. In a previous study, our group demonstrated its potential as an inhibitor of the QS system of *Pseudomonas aeruginosa* [26]. Furvina showed QS inhibitory activity, prevented *P. aeruginosa* biofilm formation and hindered the production of several virulence factors [26]. 

**Figure 1:**
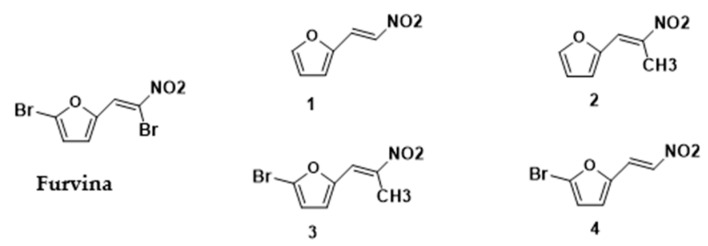
Furvina and the selected 2-nitrovinylfuran derivatives.

In this work, the effects of Furvina and selected 2-nitrovinylfuran derivatives (Figure 1) were evaluated in the *S. aureus* Agr QS system, using *S. aureus* bioreporters strains. Furvina and the best QSI was evaluated *in silico* (molecular docking simulations) and further tests were performed to assess the effects of these compounds on the prevention of biofilm setup and in the increase of biofilm susceptibility to fusidic acid (24 h-old biofilms formed in the presence of the identified QSI or Furvina).

## 2. Results

Furvina and 2-nitrovinylfuran derivatives were evaluated against planktonic *S. aureus* ALC1742 and *S. aureus* ALC1743 (Table 1) to obtain the minimum inhibitory concentration (MIC). As shown in Table 1, the only molecules that did not inhibit bacterial growth where compounds **2** and **4**. For the following QS evaluation experiments, the MIC, when found, was used as maximum concentration as well as 1000 μg/mL (the cut-off concentration tested). The cut-off concentration was used in cases of MIC lower than 1000 μg/mL, in order to test the possible effects of a concentration higher than MIC in the Agr QS as well as to corroborate the fact that the observed activity was not due to growth inhibition. 

**Figure 2:**
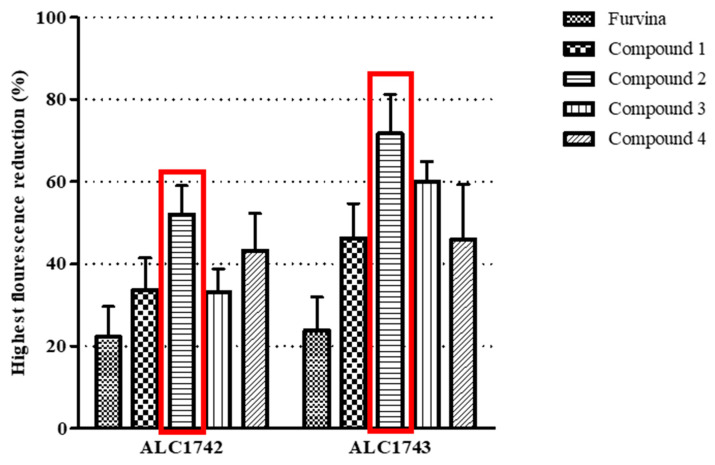
Highest fluorescence reduction percentages obtained for all the compounds tested. Almost all the compounds reached the maximum fluorescence reduction after 24 h of contact, except Furvina (the maximum reduction was after 7 h for ALC1742 and 5 h for ALC1743). The concentration in which the topmost fluorescence reduction was obtained was different between the compounds. Furvina, compound **2** and compound **4** caused the maximum reduction at 1000 μg/mL. Compound **2** reached a maximum at 1000 μg/mL for ALC1742 and 800 μg/mL for ALC1743. Compound **3** reached the maximum at 100 μg/mL for both strains. The red box highlights the compound with the highest fluorescence reduction and the one that was selected as the best QSI candidate for the following experiments. More detailed information about all the concentrations tested and for the different time-points is provided as supplementary data (Supplementary Figures S1 and S2).

Furvina and its chemically related compounds were screened to assess their effects on the *S. aureus* Agr QS system. Accordingly, and taking into account that the effect in the *S. aureus* Agr QS system is measured based on the emission of *gfp*, the highest fluorescence reduction regards the topmost inhibition of each promoter—P2 for *S. aureus* ALC1742 and P3 for *S. aureus* ALC1743, respectively [27]. As shown in Figure 2, Furvina and 2-nitrovinylfuran derivatives were able to inhibit both the promoters. However, compound **2** was that showing the highest inhibitory effect (more than 50% for both promoters).

**Figure 3:**
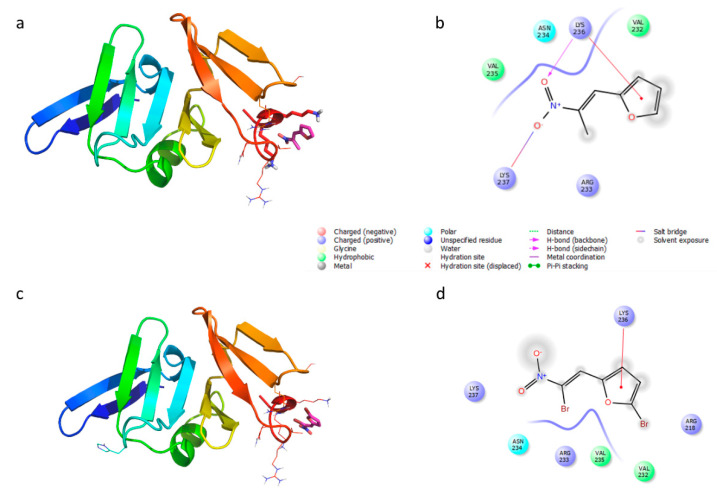
Putative binding mode on the C-terminal AgrA DNA binding domain of (**a**,**b**) compound 2 and (**c**,**d**) Furvina. The AgrA LytTR domain is shown as a rainbow-colored cartoon, ligands docked as magenta sticks, residues of the binding pocket as lines and sticks.

**Figure 4:**
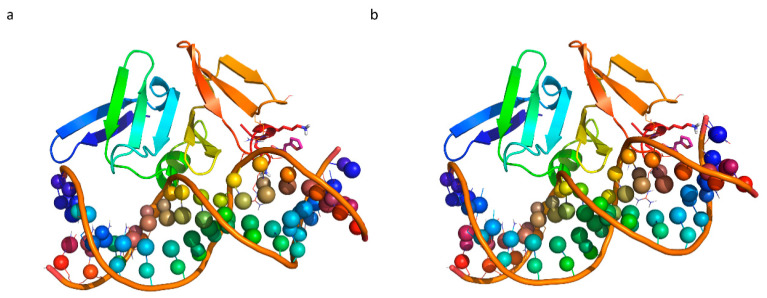
The binding of the ligand compound 2 (pink stick) would prevent the binding of promoters: Alignment of docking putative binding mode with complex AgrA-DNA promoters (**a**) 2 (RNAII) (pdb 3bs1); (**b**) 3 (RNAIII) (pdb 4xqq). The AgrA LytTR domain is shown as a rainbow-colored cartoon, ligands docked as magenta sticks, residues of the binding pocket as lines and sticks. While the DNA-promoters are depicted a rainbow-colored cartoon with the ribose as a small sphere.

Based on the in vitro data showing the interference of Furvina and compound **2** with both P2 (RNAII) and P3 (RNAIII), molecular docking simulations were carried out in the *S. aureus* Agr system to identify the preferred binding mode adopted by the compounds in the protein hydrophobic cleft pocket previously identified (LytTR domain of AgrA) [28]. Crystal structures of the apo-protein AgrA LytTR domain was downloaded from the Protein Data Bank (pdb code 4g4k [28], resolution 1.52 Å). Furvina and compound **2** were docked to the protein. Docking best poses were then energy minimized to simulate the induced fit effect of binding pocket ligand surrounding residues. Finally, the complex AgrA- compound **2** was aligned with AgrA LytTR domain complexed with P2 (RNAII) (pdb code 3bs1) [29] and P3 (RNAIII) (pdb code 4xqq) [30]. *In silico* docking experiments showed that AgrA-compound **2** complex is stabilized by several interactions with the shallow groove formed by the β10−α2 loop, particularly by the interaction with positively charged residues Lys236, Lys237, Arg233 and polar Asn234. Finally, with hydrophobic residues Val232 and Val235 (Figure 3a,b). In the case of AgrA- Furvina complex nitro group is oriented outside, which makes the complex less stabilized but still able to interact with Lys 236 with a cation-π interaction, and with hydrophobic residues Val232 and Val235 (Figure 3c,d). The binding of compound **2** would be able to interfere in the binding with P2 (RNAII) and P3 (RNAIII), as shown in Figure 4.

Furvina and compound **2** were selected to further assess their effect in the prevention of biofilm formation (24 h exposure) and to evaluate their effect on biofilm (24 h-old biofilms formed in the presence of Furvina and compound **2**)susceptibility to fusidic acid. The selected compounds (Furvina and compound **2**) as well as fusidic acid were studied for their ability to interfere with the biofilms in terms of mass removal, metabolic activity and membrane integrity (Table 2). Regarding prevention of biofilm formation, Furvina was the compound that caused the highest biofilm mass reductions (~40% for both strains at MIC and ½ MIC) (*p* < 0.05), which is ~50% higher than the values obtained for compound **2**, for both strains. In terms of metabolic inactivation, Furvina also presented the best performance with approximately 75% reduction compared to the ~40% and ~20% reduction obtained by compound **2** for ALC1742 and ALC1743, respectively (*p* < 0.0001). 

Regarding the effects of the two compounds on biofilm susceptibility to fusidic acid, the combination with compound **2** was that causing the highest potentiation of fusidic acid action. The combination of Furvina and compound **2** with fusidic acid improved biofilm mass reduction in comparison to the antibiotic alone, and no significant difference was obtained from the combination with Furvina or compound **2** (*p* > 0.05). While, for ALC1742, Furvina conjugated with fusidic acid reduced the biofilm mass in approximately 38%, compound **2** reduced in 34%, which is significantly higher than the 20% achieved by the antibiotic alone (*p* < 0.05). For ALC1743, compound **2** and fusidic acid were not able to reduce biofilm mass, while Furvina combined with the antibiotic achieved a 29% reduction. However, this reduction was not significantly different from that caused by the antibiotic alone (23%). Considering the metabolic activity reduction, compound **2** conjugated with fusidic acid caused 80% inactivation, which is significantly higher than the ~70% obtained by fusidic acid alone (*p* < 0.05 for both strains) or in combination with Furvina (*p* < 0.05 for both strains). In terms of membrane integrity of the 24-h oldbiofilms, most of the bacteria maintained their membrane intact (more than 70%), for both strains and different concentrations of compound **2** and Furvina (Table 2).

## 3. Discussion

The inappropriate use of antibiotics as well as the natural evolution and adaptation of microorganisms has led to an increase of bacterial resistance to the most commonly administered therapeutic drugs [31,32]. It is now clear that antimicrobial resistance is particularly critical when bacteria form biofilms. This is because biofilms act as a protective barrier against therapeutics and the host immune system, providing an environmental reservoirs of resistant microorganisms [33,34]. In fact, there is no pharmacotherapy effective for biofilm control [13]. The discovery and development of antibiofilm and anti-virulent agents, namely QSIs, is an emergent issue [35,36]. Inhibiting the QS system with small molecules has been considered a potential therapeutic strategy to prevent pathogenicity [34]. QS in *S. aureus* is encoded by the *agr* locus which consists of two divergent transcriptional units, RNAII and RNAIII [7]. The RNAII is responsible for the auto activation of the circuit, while RNAIII controls the expression of extracellular virulence factors, such as toxins and enterotoxins [7,9,10]. As Agr QS consists primarily of these two components—RNAII and RNAIII—the specificity of a QSI for each QS regulator needs to be evaluated accordingly. 

Even though the antimicrobial action of Furvina and effects on the QS network of *P. aeruginosa* (3-oxo-C12-HSL-based QS system) are recognized [26], no data on the effects in the QS of Gram-positive bacteria has been reported. Therefore, in this work the effects of Furvina and four related 2-nitrovinylfuran derivatives were evaluated in the QS of *S. aureus*. The study was extended to the evaluation of the effects of Furvina and the best QSI candidate on the prevention of biofilm formation and on the enhancement of biofilm susceptibility to fusidic acid.

Furvina and compound **2** were selected for further biological analysis after the QS effect screening. Furvina was selected as a baseline compound based on the recognized activity in the QS of *P. aeruginosa*, while compound **2** showed the highest fluorescence inhibition, being considered the best QSI candidate. Looking to their chemical structures, it is possible to highlight that they differ on the presence or absence of some functional groups and those differences can be associated with the increased capacity for inhibiting QS. When compared with Furvina, compound **2** has no bromine on the furan moiety and the bromine present in the styryl side chain has been replaced by a methyl group (see Figure 1). Based on this SAR analysis, it appears that the nitromethyl moiety in conjunction with a vinylfuran and the absence of bromine groups are essential for compound **2** QSI effect. In fact, it has been stated that slight changes in the chemical structure can modify the antibacterial effects of the compound [37,38]. Milhazes et al. [38] evaluated the influence of different aromatic substitution patterns in the antibacterial activity of several β-nitrostyrene and β-methyl-β-nitrostyrene derivatives. These authors studied the effect of three structural parameters in the overall stability of the molecules and correlated that with the antibacterial activity of that chemical family [38]. They investigate the relative orientation of the hydroxy and/or methoxy groups in the aromatic ring; benzene and nitro group relative to the double bound and the position of the nitrogen dioxide and methyl group (in β-methyl-β-nitrostyrene) relative to the ring and stated that slight changes in the structure conferred diverse electronic environments, contributing to differences in the antibacterial activity [38]. Scholz et al. [37] evaluated the antibacterial effect of nitrovinylfuran and some structurally related derivatives and found that the di-bromo substituted nitrovinylfuran was a potent inhibitor of MurA, a key enzyme that catalyzes the first step in peptidoglycan biosynthesis of the bacterial cell wall, and for this reason is considered an attractive target for the development of antibacterial compounds [37]. They further stated that the bromonitromethyl moiety, either alone or combined with a vinylfuran, is critical for the antibacterial and MurA inhibition properties [37]. Beyond that, Scholz et al. also studied the nitrovinylfuran derivatives stability and found that all the derivatives decompose to the corresponding aldehydes when in aqueous media [37]. This is corroborated by Allas et al. where they concluded that Furvina interconverts into a range of reaction products [39]. Despite this, both authors concluded that Furvina has reasonable long-term effects and that further research should be performed regarding the reaction products and parent molecules [37,39]. Furvina and the four related 2-nitrovinylfuran derivatives were not subject of study regarding their stability during the present experiments. 

Docking simulations demonstrated that compound **2** could interact with the protein hydrophobic cleft in the DNA binding domain (LytTR) of AgrA pocket, which has been previously identified to be important for the binding of small molecules [28]. The binding with this cleft would block the DNA binding. In fact, some residues of this area, such as Arg233, has been demonstrated to be essential for DNA binding [29]. Furthermore, it was found that this pocket to be conserved across all Staphylococcal strains. Instead, the steric hindrance of bromine in the nitro vinyl moiety of Furvina does not allow the compound to be well accommodated in the hydrophobic pocket. 

Regarding biofilm prevention, this study demonstrates that Furvina had a higher antimicrobial and antibiofilm activity than compound **2**. This result was excepted as the mode of action of Furvina involves the reaction with the cysteine residues of MurA, a protein that is crucial for peptidoglycan synthesis, which in turn is the main component of the bacterial cell wall [37]. Additionally, this study indicates that compound **2** interferes with *S. aureus* Agr QS and increases *S. aureus* biofilm susceptibility to fusidic acid. These results corroborate a previous study showing that QS signaling molecules are involved in many aspects of biofilm dynamics (e.g., heterogeneity, architecture, stress resistance, maintenance, and sloughing) [40]. Once a blockage to these molecules occurs, an increase in biofilm susceptibility to antibiotics and host defenses will happen, favoring the use of low doses of antibiotics and leading to an easier biofilm eradication [40]. Even though, the mechanism of synergy between fusidic acid and compound **2** remains to be understood. Fusidic acid inhibits protein synthesis in Gram-positive bacteria, binding to elongation factor G (EF-G) and locking it to the ribosome [41]. Despite the lack of knowledge of the joint action of these compounds there are advantages in using combinatorial approaches. The overall results showed that almost all the compounds caused modest effects on the membrane integrity, without affecting cell growth, which is in accordance with Borges et al. [26]. This is particularly noteworthy, as QSIs must be effective in treating bacterial infections by rendering the pathogen avirulent or less fit to survive within the host, not by inducing toxicity in the bacteria or death [27].

Fusidic acid is a drug that has excellent bioavailability and skin penetration [42]. Furthermore, it is a drug that is metabolized in the liver and excreted in the bile, which could potentially reduce the risk of toxicity with co-administration. Even though resistance to fusidic acid started to appear due to the unrestricted use of this antibiotic, its combination with a QSI could potentially make it effective again for treating skin infections. Moreover, the *agr* dysfunctional bacteria are less prone to be transmitted between patients, suggesting that whereas *agr* mutants arise with the use of these combinatorial approaches, they are unlikely to have a selective advantage over wild-type bacteria [43,44]. Importantly, infections containing *agr* mutants are mostly associated with bacteremia and not with acute skin infection in immunocompetent individuals [44,45].

Overall, this study proposes that the virulence of staphylococci is affected by the inhibition of the Agr QS system, which is in agreement with previous findings [46,47,48,49]. Indeed, the Agr QS system is assumed to be associated to the overall virulence of staphylococci and *agr* expression appears to contribute to Staphylococcal pathogenesis in several infection models [46,47,48,49]. This is an important step for biofilm control as there is a huge need to develop new therapeutic methods, specially anti-virulence therapies that target key regulators involved in the establishment and propagation of infections [50].

## 4. Materials and Methods

### 4.1. Synthesis of Furvina and 2-Nitrovinylfuran Derivatives

Furvina and 2-nitrovinylfuran derivatives 1–4 were synthesized as previously described [26,44,45]. The structural elucidation was performed by spectroscopic techniques (NMR and MS) and the data is in accordance to the literature [26,51,52].

### 4.2. Preparation of 2-Nitrovinylfuran Derivatives Stock Solutions

Furvina and 2-nitrovinylfuran derivatives 1–4 solutions were prepared in dimethyl sulfoxide (DMSO, 100%, *v*/*v*; Fisher Scientific, Loughborough, UK) under sterile conditions. Serial dilutions of the compounds, from 1000 μg/mL to 6.25 μg/mL, were prepared when needed. The percentage of DMSO never exceeded 10% (*v*/*v*) of the final volume. Fusidic acid was purchased from Sigma-Aldrich and used according to the manufacturer’s recommendations.

### 4.3. Microorganisms and Culture Conditions

Two *S. aureus* strains—ALC1742 with RNAII promoter and ALC1743 with RNAIII promoter [53] were used for the studies of: the effect of Furvina and 2-nitrovinylfuran derivatives on *S. aureus* Agr QS and the effect of the best QSI’s candidate on *S. aureus* biofilm formation and susceptibility to fusidic acid. These strains were constructed by Xiong et al. in which it was used a GFP reporter gene system [53]. The DNA constructs containing either RNAII and RNAIII promoter sequences of *agr* were cloned upstream of the promoterless GFP gene in the shuttle plasmid [53]. The recombinant plasmids were then electroporated in the *S. aureus* [53]. Some preliminary results indicate that the expression of GFP is dependent on the strength of the promoter inserted [53].

The *S. aureus* strains were inoculated aerobically in Tryptic Soy Broth (TSB; Merck, Darmstadt, Germany) at 37 °C. Regarding the study of the effect of the selected compounds in QS, bacteria were cultured without shaking to prevent *gfp* expression, the parameter measured in this experiment [53]. However, for the biofilm formation study the agitation was used at 150 rpm (AGITORB 200, Aralab, Rio de Mouro, Portugal).

### 4.4. Determination of the Minimum Inhibitory Concentration (MIC)

The MIC of Furvina, 2-nitrovinylfuran derivatives 1–4 and fusidic acid were determined based on the microdilution method described by Borges et al. [54]. This method comprises an overnight growing of bacteria in TSB and then an adjustment of the optical density (OD) to 0.132 ± 0.02 (λ = 600 nm) (~3 × 10^8^ CFU/mL). Then, a volume of 180 μL of this cell suspension was added to a sterile, 96-well flat, clear bottomed polystyrene (PS) microtiter plates (Orange Scientific, Braine—l’Alleud, Belgium), already containing 20 μL of compound at concentrations from 6.25 to 1000 μg/mL. Regarding fusidic acid, the range of concentrations tested were 0.0625–32 μg/mL. At the end, the volume of each compound never exceeded 10% (*v*/*v*) of the well volume. Microtiter plates were then incubated for 24 h at 37 °C at 150 rpm. Absorbance measurements were performed in the beginning (t = 0 h) and in the end (t = 24 h) of the incubation period using a microplate reader (Synergy HT, Biotek, Winooski, VT, USA). Cell suspensions with and without DMSO were used as controls. MIC was set as the lowest concentration of the compound at which the final OD was equal or lower than the initial OD (cell growth inhibition). This test was performed three times, with six replicates.

### 4.5. Detection of Quorum Sensing Inhibition

The interference of Furvina and 2-nitrovinylfuran derivatives in the QS system of *S. aureus* ALC1742 and ALC1743 was evaluated based on Xiong et al. [53]. Bacteria grown overnight at 37 °C in TSB was adjusted to an OD of 0.04 ± 0.02 (~1 × 10^7^ CFU/mL) at 620 nm. A volume of 180 μL of the cell suspensions was added to a sterile, 96-well flat-bottomed, polystyrene (PS) microtiter plates (Thermo scientific, Roskilde, Denmark) containing 20 μL each compound at different concentrations (6.25–1000 μg/mL). At selected time points (1, 3, 5, 7, and 24 h), the emitted fluorescence (ʎexcitation: 485–12 nm and ʎ_emission_: 510–10 nm) was measured using a microtiter plate reader (Fluorstar Omega; BMG Labtech; Ortenberg, Germany). The effect on the promoter was measured as fluorescence reduction of the cells when in contact with a specific compound compared to cells in contact with 10% (*v*/*v*) DMSO. All experiments were performed three times, with six replicates.

### 4.6. Molecular Docking

(a) Ligands Preparation

Theoretical 3D models of compounds were built by means of Maestro GUI. Ligand’s global minimum energy conformation was determined by molecular mechanics conformational analysis performed with Macromodel software version 9 [55]. The geometry was optimized by MMFFs (Merck molecular force fields) [56] and GB/SA water implicit solvation model [57] using Polak-Ribier Conjugate Gradient (PRCG) method, 5000 iterations and a convergence criterion of 0.05 kcal/(mol Å). All other parameters were left as default.

(b) Protein Preparation

The protein structure was obtained from the PDB web site with pdb code 4g4k and was prepared by using the Protein Preparation Wizard module [28]. Water molecules were removed.

(c) Docking simulations

Three protocols were compared: Glide-SP [58], Glide-XP [58], Glide Quantum-Mechanical Polarized Docking (QMPL)-SP [59]. All protocols gave a similar binding mode as best pose which was then selected for energy minimization. The minimization protocol considered 10,000 steps of the Polak-Ribier conjugate gradient (PRCG) minimization method using OPLS_2005 force field [60]. The optimization process was performed up to the derivative convergence criterion equal to 0.05 kcal/(mol*Å)^−1^. Depictions were obtained with Pymol (PyMOL Molecular Graphics System, Version 1.7.2.1 Schrödinger, LLC) and Maestro.

### 4.7. Effect of Furvina and 2-Nitrovinylfuran Derivatives on the Prevention of Biofilm Formation

Biofilms were developed according to the modified microtiter plate assay proposed by Stepanovic et al. [61]. Furvina and the best QSI candidate were the compounds evaluated in terms of *S. aureus* ALC1742 and *S. aureus* ALC1743 biofilm formation. For at, 96-well flat, clear bottomed PS microtiter plates were filled with 180 μL of cells suspension (~1 × 10^7^ CFU/mL) and 20 μL of the compounds at MIC and ½ MIC. Bacterial suspensions with DMSO and without compounds were used as controls. The plates were incubated at 37 °C and 150 rpm for 24 h (biofilm formation). After the incubation period, the content of each well was discarded and washed twice with sterile phosphate buffer saline (PBS, pH = 7.4). Microtiter plates were analyzed in terms of biomass quantification by crystal violet staining (CV; Merck, Darmstadt, Germany), metabolic activity by alamar blue (AB; Merck, Darmstadt, Germany) staining and also in terms of membrane integrity using the Live/Dead Baclight kit.

(a) Biomass quantification of biofilm cells by crystal violet staining

Biofilm mass quantification by CV staining was assessed according to Borges et al. [62]. After the 24 h incubation period, the content of each well was discarded, and the wells washed with 250 μL of sterile PBS (pH = 7.4), to remove all non-adherent and weakly adhered bacteria. The remaining adhered bacteria were fixed with 250 μL of 96% (*v*/*v*) commercial ethanol for 15 min. The content of the microtiter plates was again rejected, and the plate was left to dry. Then, the fixed bacteria were stained for 5 min with 200 μL of 1% CV solution. After that period, the excess of stain was gently withdrawn. Afterwards, the dye bound to the adherent cells was resolubilized with 200 μL of 33% (*v*/*v*) glacial acetic acid (Fisher Scientific) and the absorbance was measured at 570 nm using a microtiter plate reader. All tests were performed in triplicate with six replicates. The results were presented in terms of percentage of biofilm mass removal when exposed to the different compounds at the respective concentrations according to Equation (1):

(1) $$\%\;\mathrm{BR}\;=\;\frac{{\mathrm{OD}}_{\mathrm{control}}-{\mathrm{OD}}_{\mathrm{compound}}}{{\mathrm{OD}}_{\mathrm{control}}}\;\times 100$$

where %BR is the percentage of biofilm removal, OD_control_ is the OD (λ = 570 nm) for biofilms not exposed and OD_compound_ is the OD (λ = 570 nm) for biofilms exposed to the selected compounds.

(b) Metabolic activity quantification of biofilm cells by alamar blue staining

The quantification of the metabolic activity of biofilm cells was performed according to Borges et al. [62,63]. Briefly, the microtiter plate content was removed and the wells washed with 250 μL of sterile PBS (pH = 7.4), to remove all non-adherent and weakly adhered bacteria. For the staining procedure, 190 μL of fresh TSB was added to the microtiter plates, as well as 10 μL of alamar blue indicator solution (0.4 mM). Plates were then incubated for 20 min in the dark and at 37 °C. Fluorescence was measured at λ_excitation_ = 570 nm and λ_emission nm_ = 590 nm in a microtiter plate reader (Fluorstar Omega; BMG Labtech; Ortenberg, Germany). All tests were performed at least three time with six replicates. Results were obtained in terms of percentage of biofilm metabolic activity reduction when exposed to the different compounds. The percentage of biofilm metabolic activity reduction was determined according to the following Equation (2):

(2) $$\%\;\mathrm{BAR}=\;\frac{{\mathrm{FI}}_{\mathrm{control}}-{\mathrm{FI}}_{\mathrm{compound}}}{{\mathrm{FI}}_{\mathrm{control}}}\;\times \;100$$

where %BAR is the percentage of biofilm metabolic activity reduction, FI_control_ is the fluorescence intensity of not exposed biofilms and FI_compound_ is the fluorescence intensity for biofilms exposed to the selected compounds.

(c) Membrane integrity of biofilm cells

The membrane integrity of *S. aureus* biofilm cells was evaluated by the Live/Dead Baclight kit (Invitrogen-Molecular Probes, The Netherlands) after exposure to the selected compounds. This kit is composed by two nucleic acid binding stains: SYTO 9™ and propidium iodide (PI). Briefly, biofilm cells, previously obtained by scraping of the wells of microtiter plate were diluted (1:10) in PBS (pH = 7.4). Then, a volume of 300 μL of each diluted bacterial suspension was filtered through a Nucleopore black polycarbonate membrane (Whatman, UK; pore size 0.22 μm) and then stained with 250 μL of SYTO 9™ and 50 μL of PI [54]. This mixture was left to react for 7 min in the dark and then observed in the microscope. The membrane was then placed in the microscope slide and observed in a LEICA DMLB2 epifluorescence microscope (Leica Microsystems Ltd., Wetzlar, Germany) coupled with a Leica DFC300 FX camera (Leica Microsystems Ltd.), using 100× oil immersion fluorescence objective. The number of membrane damaged/not damaged cells per mL in the sample were presented according to the following equation:

(3) $$N\;=\;\frac{n\times A}{B\times V}\;\times D$$

where N is the number of cells per mL, n is the average number of cells per microscopic field, A is the membrane area (cm^2^), B is the microscopic field area (cm^2^), V is the volume of the filtered sample (mL) and D is the dilution factor.

The percentage of intact cells were presented according to the following equation:

(4) $$\%\;\mathrm{Intact}\;\mathrm{cells}\;=\;\frac{N_{\mathrm{green}}}{N_{\mathrm{total}}}\;\times \;100$$

The results were presented as percentage of cells stained with SYTO 9^TM^ as a function of concentration.

### 4.8. Effect of Furvina and the Best QSI Candidate on Biofilm Susceptibility to Fusidic Acid

To evaluate the effect of the best compound on biofilm susceptibility to fusidic acid, biofilms were developed in the presence of 1000 μg/mL of the compound (since no MIC for this compound was detected within the concentrations tested). Bacterial suspensions with DMSO and without compound were used as controls. Besides that, Furvina was also tested as baseline compound for comparative purposes. After 24 h incubation at 37 °C and 150 rpm, 24 h-old biofilms were washed twice with PBS (pH = 7.4) and exposed to fusidic acid at 10× MIC. The plates were incubated for 24 h at 37 °C and 150 rpm. The biofilms were analyzed in terms of biomass, metabolic activity and membrane integrity as described above.

### 4.9. Statistical Analysis

Statistical analysis was performed using GraphPad Prism software version 5.01 (GraphPad Software Inc., San Diego, CA, USA). One-way ANOVA and multiple comparisons were used to test the significance based on a confidence level of ≥95% (*p* < 0.05, statistically significant). All experiments were performed in duplicate with at least three replicates for each condition tested.

## Figures and Tables

**Table 1:** MIC of the compounds for both *S. aureus* ALC1742 and *S. aureus* ALC1743 strains.

Chemical Structure	Designation	MIC (µg/mL)	
		*S. aureus* ALC1742	*S. aureus* ALC1743
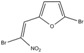	Furvina	100	100
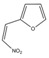	Compound **1**	500	400
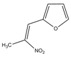	Compound **2**	>1000	>1000
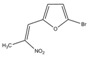	Compound **3**	400	400
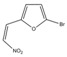	Compound **4**	>1000	>1000
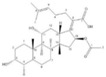	Fusidic acid	0.25	0.25

**Table 2:** Percentage of biomass and metabolic activity reduction, and membrane intact biofilm cells of *S. aureus* (ALC1742 and ALC1743) formed in the presence of Furvina and compound **2**, at MIC and ½ MIC, or the highest concentration tested (HCT) and ½ HCT. The top part of the Table refers to the prevention of biofilm setup and the bottom part concerns the effects against 24 h-old biofilms (formed in the presence of Furvina and compound **2**) to fusidic acid (- * no biomass reduction). Mean values ± standard deviation are illustrated.

Compound	Strain	Concentrations (μg/mL)	Biomass Reduction (%)	Metabolic Activity Reduction (%)	Intact Cells (%)
Furvina	ALC1742	MIC (100)	42.0 ± 8.0	75.1 ± 5.0	85.4 ± 8.1
		½ MIC (50)	37.5 ± 7.5	66.4 ± 8.0	86.1 ± 9.8
	ALC1743	MIC (100)	44.6 ± 1.7	78.6 ± 1.0	75.0 ± 18.6
		½ MIC (50)	41.2 ± 5.4	77.8 ± 1.7	84.4 ± 14.6
Compound 2	ALC1742	HCT of compound **2** (1000)	19.0 ± 3.0	42.1 ±7.4	95.0 ± 3.0
		½ HCT of compound **2** (500)	17.5 ± 2.9	41.6 ± 8.5	93.6 ± 5.7
	ALC1743	HCT of compound 2 (1000)	17.3 ± 3.3	28.6 ± 8.6	94.0 ± 2.7
		½ HCT of compound **2** (500)	21.5 ± 1.0	17.8 ± 4.1	93.7 ± 1.9
Fusidic acid	ALC1742	10× MIC (2.5)	19.97 ± 7.9	71.0 ± 5.9	91.5 ± 3.6
	ALC1743	10× MIC (2.5)	22.7 ± 8.8	70.8 ± 6.6	87.8 ± 6.6
Fusidic acid + Compound 2	ALC1742	10× MIC fusidic acid (2.5) + HCT of compound **2** (1000)	33.7 ± 4.0	81.7 ± 5.8	66.9 ± 8.3
	ALC1743	10× MIC fusidic acid (2.5) + HCT of compound **2** (1000)	- *	82.3 ± 4.0	81.1 ± 4.2
Fusidic acid + Furvina	ALC1742	10× MIC fusidic acid (2.5) + MIC (100)	38.0 ± 14.4	72.6 ± 4.2	90.2 ± 4.7
	ALC1743	10× MIC fusidic acid (2.5) + MIC (100)	28.9 ± 4.7	74.3 ± 4.3	85.6 ± 5.0

## Data Availability

Data is contained within the article or supplementary material.

## Supplementary Materials

Supplementary materials can be found at https://www.mdpi.com/1422-0067/22/2/613/s1. Figure S1: Quorum-sensing interference screening using Furvina and all the structurally related compounds at different time-points. Figure S2: Quorum-sensing interference screening using Furvina and all the structurally related compounds at different time-points.

## Abbreviations

QS	Quorum sensing
QSI	Quorum sensing inhibitor
SSTI	Skin and soft tissue infections
Ai	Autoinducers
HSL	N-acyl-homoserine lactone
AIP	Autoinducer peptides
Agr	Accessory gene regulator
EPS	Extracellular polymeric substances
MIC	Minimum inhibitory concentration
HCT	Highest concentration tested
DMSO	Dimethyl sulfoxide
TSB	Tryptic soy broth
Gfp	Green fluorescence protein
OD	Optical density
PS	Polystyrene
CV	Crystal violet
PBS	Phosphate buffer saline
PI	Propidium iodide
