# Supplementary material for: 2-(2-Methyl-2-nitrovinyl)furan but Not Furvina Interfere with Staphylococcus aureus Agr Quorum-Sensing System and Potentiate the Action of Fusidic Acid against Biofilms
Source: Int J Mol Sci. 2021 Jan 9;22(2):613. doi: 10.3390/ijms22020613 (PMC7827229; doi:10.3390/ijms22020613)
Supplement: Supplementary file 1 [file ijms-22-00613-s001.pdf]

Supplemental material

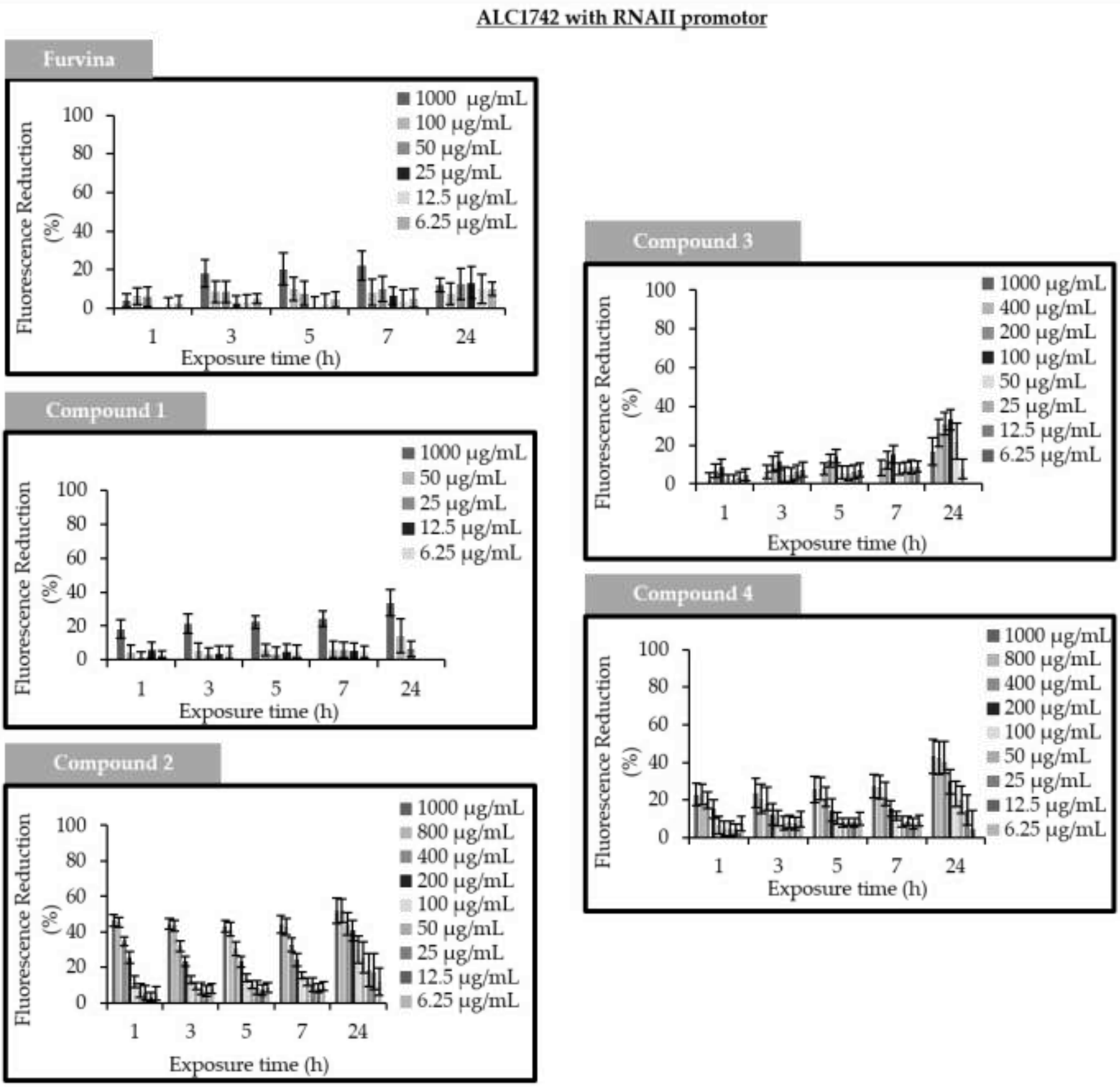

Figure S1 – Quorum-sensing interference screening using Furvina and all the structurally related compounds at different time-points. The interference was measured as the fluorescence emission reduction of ALC1742 with a RNAII promotor caused by the compounds. Mean values ± standard deviation are illustrated in the figure.

ALC1743 with RNAIII promotor

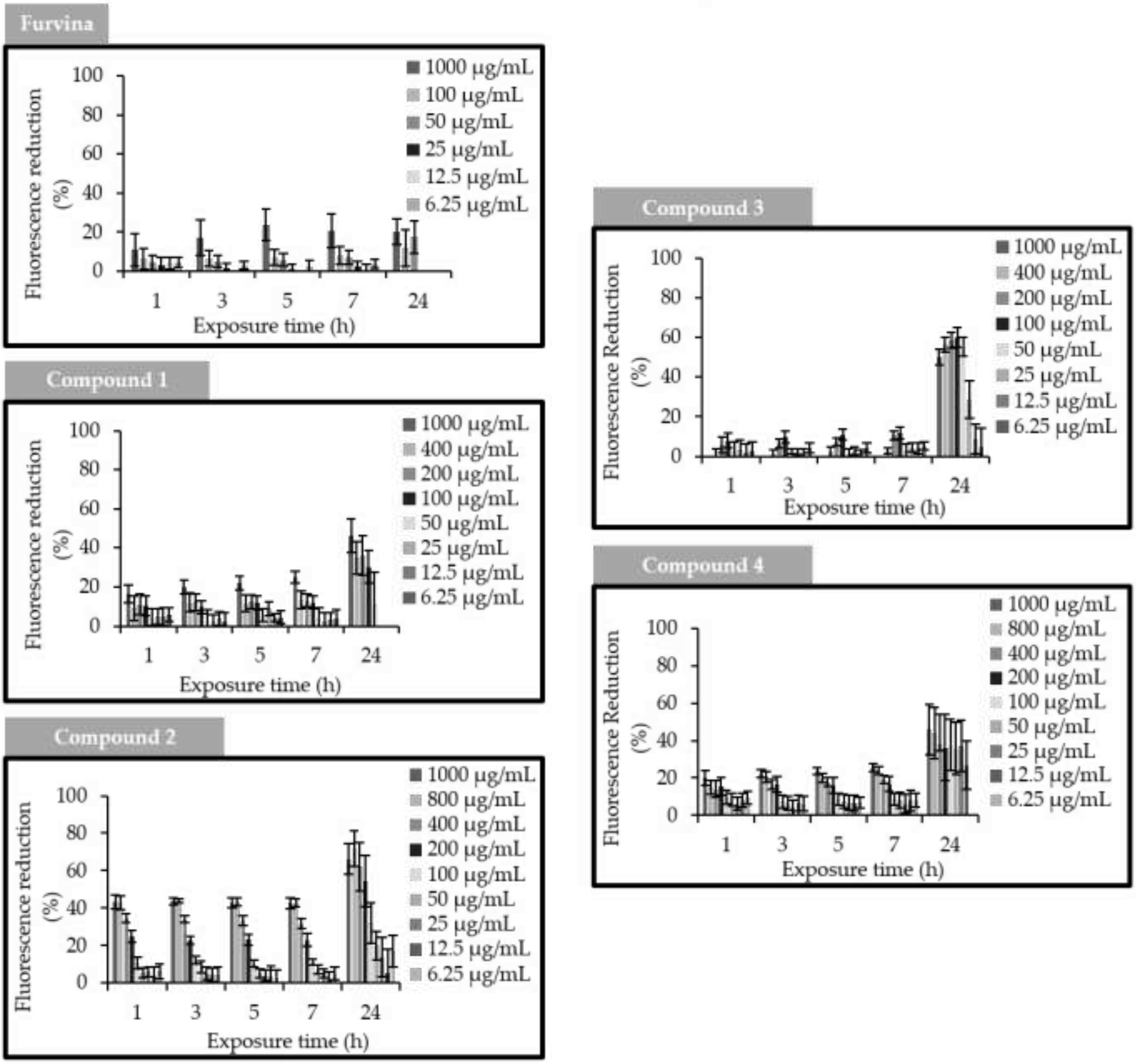

Figure S2 – Quorum-sensing interference screening using Furvina and all the structurally related compounds at different time-points. The interference was measured as the fluorescence emission reduction of ALC1743 with a RNAIII promotor caused by the compounds. Mean values ± standard deviation are illustrated in the Figure.
